# Plasma Citrate Levels Are Inversely Associated with Estimated Muscle Mass and Strength in Liver Transplant Recipients

**DOI:** 10.3390/ijms27114809

**Published:** 2026-05-27

**Authors:** Yakun Li, Adrian Post, Mateo Chvatal Medina, Caecilia S. E. Doorenbos, Margery A. Connelly, Han Moshage, Stephan J. L. Bakker, Vincent E. de Meijer, Robin P. F. Dullaart

**Affiliations:** 1 Department of Gastroenterology and Hepatology, University Medical Center Groningen, University of Groningen, P.O. Box 30.001, 9700 RB Groningen, The Netherlands; 2 Department of Internal Medicine, Division of Nephrology, University Medical Center Groningen, University of Groningen, P.O. Box 30.001, 9700 RB Groningen, The Netherlands; 3 Labcorp, 100 Perimeter Park, Morrisville, NC 27560, USA; 4 Department of Surgery, Division of Hepato-Pancreato-Biliary Surgery and Liver Transplantation, University Medical Center Groningen, University of Groningen, P.O. Box 30.001, 9700 RB Groningen, The Netherlands; 5 Department of Internal Medicine, Division of Endocrinology, University Medical Center Groningen, University of Groningen, P.O. Box 30.001, 9700 RB Groningen, The Netherlands

**Keywords:** liver transplantation, liver transplant recipients, citrate, muscle mass, muscle strength, physical performance, mitochondrial dysfunction

## Abstract

Reduced muscle mass and strength are highly prevalent in liver transplant recipients (LTRs). Citrate, a key intermediate of the tricarboxylic acid cycle, may adversely relate to muscle health through disturbances in mitochondrial energy metabolism and metabolic flexibility. We aimed to investigate the association of plasma citrate levels with estimated muscle mass, strength, and physical performance in LTRs. We included stable LTRs from the TransplantLines Biobank and Cohort Study at least 1 year after transplantation. Muscle mass, strength, and physical performance were assessed using the 24 h urinary creatinine excretion rate divided by height squared (creatinine excretion rate index, CERI), handgrip strength, sit-to-stand (STS) and Timed Up and Go (TUG) tests. Associations of plasma citrate with muscle-related parameters were examined using Spearman correlation and linear regression analyses. A total of 501 LTRs were included (median age 59 [49–67] years; 58.7% men) at a median of 14.0 (7.4–22.5) years post-transplantation. Spearman correlation analyses showed that plasma citrate levels were inversely correlated with CERI (ρ= −0.226, *p* < 0.001) and handgrip strength (ρ= −0.211, *p* < 0.001). Additionally, plasma citrate levels were positively correlated with STS time (ρ = 0.170, *p* = 0.019) and TUG time (ρ = 0.203, *p* = 0.006). In linear regression analyses, higher plasma citrate levels were associated with lower CERI and lower handgrip strength and with longer STS and TUG time. In multivariable linear regression analyses, plasma citrate remained independently associated with CERI (fully adjusted standardized β= −0.14, *p* = 0.001) and handgrip strength (fully adjusted standardized β= −0.11, *p* = 0.010), whereas associations with physical performance measures were no longer significant after adjustment. CERI mediated 45.8% of the association between citrate and handgrip strength. In conclusion, higher plasma citrate levels are independently associated with lower estimated muscle mass and strength in LTRs. Circulating citrate may serve as a potential biomarker of post-transplant muscle impairment risk.

## 1. Introduction

Liver transplantation has substantially improved survival for patients with end-stage liver disease, but long-term morbidity remains considerable [1,2,3,4]. Impaired skeletal muscle health, manifesting as reduced muscle mass, decreased strength, and diminished physical performance, is common in liver transplant candidates and persists among liver transplant recipients (LTRs) [5,6]. This is clinically important, as post-transplant muscle loss and dysfunction contribute to frailty and functional limitations and are independently associated with adverse clinical outcomes after transplantation, including mortality [7,8,9].

Post-transplant muscle impairment is likely multifactorial, driven by persistent metabolic stress and inflammation, age-related decline in muscle mass, impaired nutritional status, reduced physical activity, as well as the metabolic effects of long-term immunosuppression [10,11,12]. These factors may converge on disrupted protein turnover, mitochondrial dysfunction with impaired energetics and substrate utilization, and dysfunction of the neuromuscular unit, thereby contributing to muscle wasting and functional decline [13,14]. In this context, circulating metabolites related to mitochondrial metabolism may provide biologically informative markers of muscle phenotype in LTRs.

Citrate is a central intermediate of the tricarboxylic acid (TCA) cycle and acts as a metabolic hub connecting mitochondrial oxidative metabolism to cytosolic acetyl-CoA production and downstream lipid biosynthesis via ATP-citrate lyase [15]. Circulating citrate may therefore reflect alterations in mitochondrial flux and whole-body metabolic state [16]. Recent advances in high-throughput nuclear magnetic resonance (NMR)-based metabolomics enable robust and precise quantification of citrate in large cohorts, with reported analytical performance supporting its use as a scalable biomarker [17,18]. Plasma citrate is elevated in severe hypothyroidism and liver cirrhosis, an effect that is reversed after treatment of hypothyroidism and liver transplantation, respectively [18,19]. High plasma citrate is also associated with mortality in patients with end-stage liver disease and type 2 diabetes [20]. These findings corroborate the idea that elevated circulating citrate may serve as a proxy of mitochondrial dysfunction. However, the relationship between plasma citrate and skeletal muscle phenotypes (including muscle mass and function) in LTRs has not yet been documented.

Therefore, we aimed to investigate the cross-sectional associations between plasma citrate concentrations and (i) muscle mass estimated by the 24 h urinary creatinine excretion rate index (CERI), (ii) muscle strength assessed by handgrip strength, and (iii) physical performance assessed by the Sit-to-Stand (STS) and Timed Up and Go (TUG) tests in clinically stable LTRs participating in the TransplantLines cohort.

## 2. Results

### 2.1. Baseline Characteristics

A total of 501 LTRs were included (median age 59 [IQR 49–67] years; 58.7% men), at a median of 14.0 (IQR 7.4–22.5) years post-transplantation. Participants were categorized into quartiles according to plasma citrate concentrations (Q1 < 101 µmol/L; Q2 101–121 µmol/L; Q3 121–148 µmol/L; Q4 > 148 µmol/L). Baseline characteristics by citrate quartiles are presented in Table 1.

Across increasing citrate quartiles, significant differences were observed in age, sex distribution, and body mass index (BMI) (all *p* < 0.05). Higher citrate levels were also associated with differences in alcohol intake, hypertension prevalence, use of oral glucose-lowering drugs and antihypertensives, immunosuppressive regimens, and laboratory parameters including aspartate aminotransferase (AST), alkaline phosphatase (ALP), albumin, high-sensitivity C-reactive protein (hs-CRP), total cholesterol, triglycerides, hemoglobin, estimated glomerular filtration rate (eGFR), total branched-chain amino acids (BCAA), and GlycA (all *p* < 0.05).

With respect to muscle-related parameters, CERI decreased across citrate quartiles (*p* < 0.001) and handgrip strength was progressively lower in higher quartiles (*p* < 0.001). TUG performance also differed across quartiles (*p* = 0.040), whereas no significant differences were observed for the STS test (*p* = 0.082).

### 2.2. Correlation of Muscle Mass and Strength Parameters

**Figure 1:**
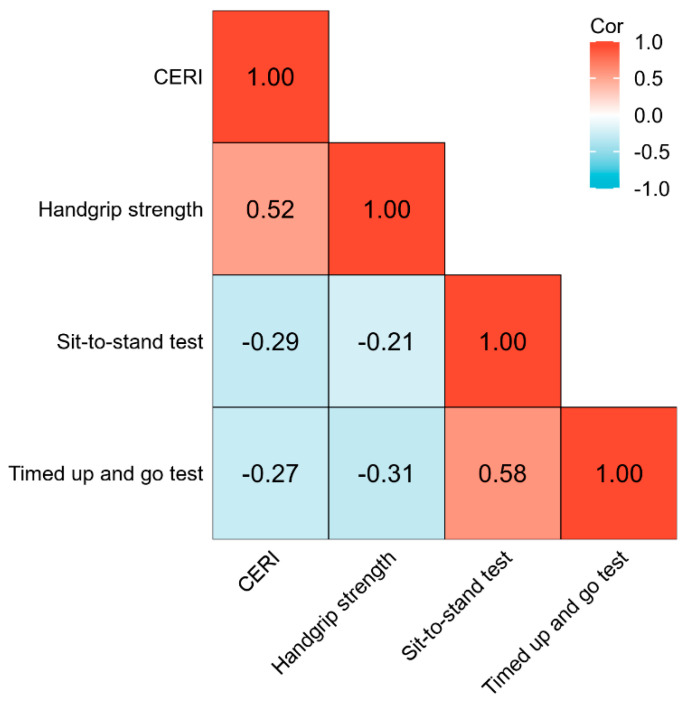
Correlation heatmap of muscle mass, strength, and physical performance measures. Numbers indicate Spearman’s rank correlation coefficients (ρ). CERI, 24 h urinary creatinine excretion rate index.

To evaluate the consistency among the different functional tests, we analyzed the correlations between parameters reflecting estimated muscle mass and strength. As shown in Figure 1, the CERI showed a significant positive correlation with handgrip strength (ρ = 0.52, *p* < 0.001). Conversely, CERI was inversely correlated with both the STS time (ρ = −0.29, *p* < 0.001) and the TUG time (ρ = −0.27, *p* = 0.0003). Higher handgrip strength was significantly associated with shorter (better) performance times in the STS (ρ = −0.21, *p* = 0.003) and TUG tests (ρ = −0.31, *p* < 0.001). Furthermore, a strong positive correlation was observed between the STS and TUG tests (ρ = 0.58, *p* < 0.001).

### 2.3. Association Between Plasma Citrate and Muscle Parameters

**Figure 2:**
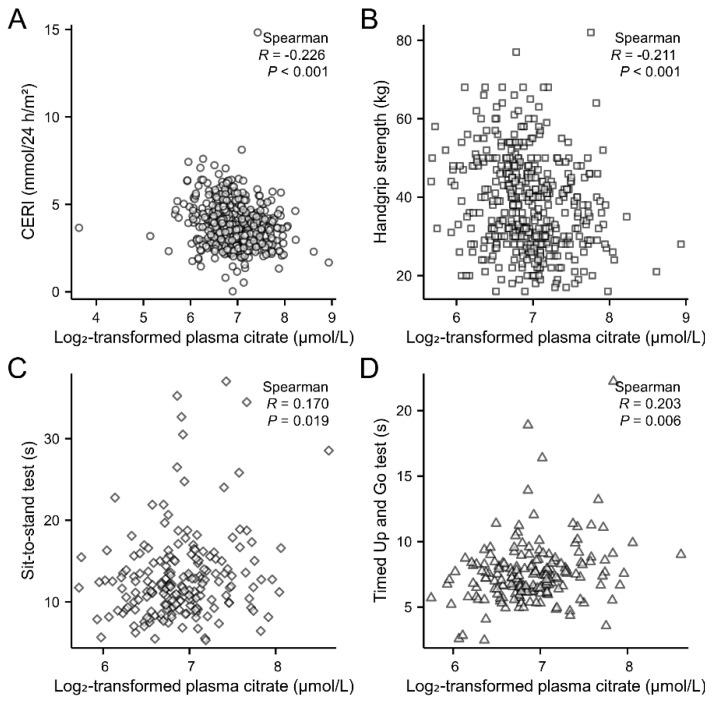
Associations between plasma citrate levels and (**A**) CERI (24 h urinary creatinine excretion rate index), (**B**) handgrip strength, (**C**) sit-to-stand (STS) test time, and (**D**) Timed Up and Go (TUG) test time. Spearman’s rank correlation coefficients (ρ) and *p*-values are shown in each panel.

Spearman correlation analyses showed that plasma citrate levels were inversely correlated with CERI (ρ = −0.226, *p* < 0.001) and handgrip strength (ρ = −0.211, *p* < 0.001) (Figure 2). In addition, higher plasma citrate levels were positively correlated with STS test time (ρ = 0.170, *p* = 0.019) and TUG time (ρ = 0.203, *p* = 0.006), indicating poorer physical performance at higher citrate levels.

The independent associations between plasma citrate levels and measurements of estimated muscle mass and strength were further evaluated using linear regression analyses (Table 2). In the crude model (Model 1), higher plasma citrate levels were significantly associated with lower CERI (Std. β = −0.19; 95% CI: −0.28, −0.10; *p* < 0.001) and reduced handgrip strength (Std. β = −0.19; 95% CI: −0.29, −0.10; *p* < 0.001). Furthermore, higher citrate levels were associated with longer completion times for the STS (*p* = 0.005) and TUG tests (*p* = 0.001). These inverse associations remained robust for CERI and handgrip strength in multivariable analyses. After adjustment for age, sex, and BMI (Model 2) and an additional adjustment for alcohol intake, hypertension, diabetes, eGFR, AST, hs-CRP, GlycA and BCAA in Model 3, plasma citrate remained independently associated with CERI (Model 3: Std. β = −0.11, *p* = 0.014) and handgrip strength (Model 3: Std. β = −0.14, *p* = 0.001). In the fully adjusted model (Model 4), which additionally included immunosuppressant use and time since transplantation, these associations persisted for CERI (Std. β = −0.14, *p* = 0.001) and handgrip strength (Std. β = −0.11, *p* = 0.010). However, the associations with STS and TUG performance were attenuated and no longer statistically significant in the fully adjusted model (STS: Std. β = 0.10, *p* = 0.242; TUG: Std. β = 0.11, *p* = 0.249). No significant effect modification by sex, age, hypertension, or diabetes status was observed (all *p* for interaction > 0.05).

The associations between citrate quartiles and the odds of below-median estimated muscle mass/strength (and above-median performance time) are shown in Supplementary Table S1. In the univariable analysis, participants in the highest citrate quartile (Q4) exhibited significantly higher odds of having below-median CERI (OR 2.89, 95% CI: 1.73, 4.82, *p* < 0.001), low handgrip strength (OR 3.12, 95% CI: 1.08, 9.02, *p* = 0.036), and above-median TUG time (OR 3.78, 95% CI: 1.5, 9.53, *p* = 0.005) compared to those in Q1. After adjustment for age, sex, BMI, and other potential confounders, the association remained significant only for below-median CERI (Q4 vs. Q1: OR 2.10, 95% CI: 1.06, 4.19, *p* = 0.034), whereas associations with other outcomes were attenuated.

**Figure 3:**
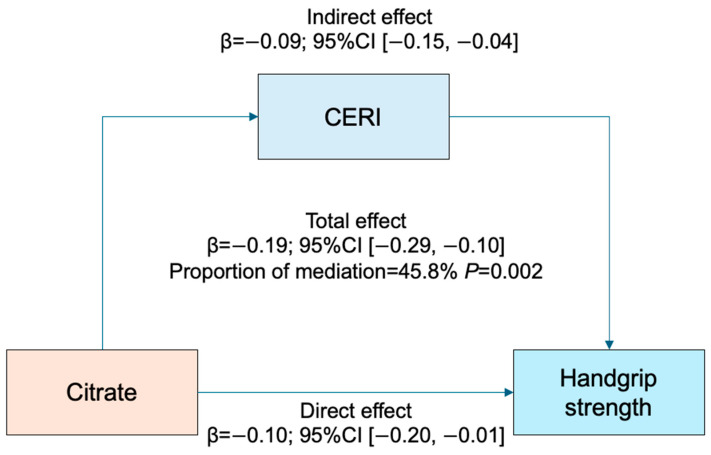
Mediation analysis of the association between plasma citrate and handgrip strength via estimated muscle mass (CERI). CERI: 24 h urinary creatinine excretion rate index.

Mediation analysis (Figure 3) showed that CERI significantly mediated the association between plasma citrate and handgrip strength (indirect effect −0.09; 95% CI −0.15 to −0.04; *p* = 0.002), accounting for 45.8% of the total effect. The mediation effect remained significant in sensitivity analyses using height-adjusted handgrip strength (proportion of mediation 42.8%, *p* < 0.001). In addition, the indirect effects were not statistically significant for either the STS (indirect effect 0.03; 95% CI −0.00 to 0.09; *p* = 0.078) or the TUG time (indirect effect 0.02; 95% CI −0.00 to 0.10; *p* = 0.088).

## 3. Discussion

To our knowledge, the present study is the first to investigate the association between plasma citrate levels and muscle-related parameters specifically in LTRs. We found that higher plasma citrate concentrations were independently and inversely associated with both muscle mass, estimated by CERI, and muscle strength, assessed by handgrip strength. Furthermore, higher plasma citrate levels were also associated with poorer physical performance, although these associations were attenuated after multivariable adjustment. Taken together, our findings indicate an independent association between higher plasma citrate levels and lower estimated muscle mass and strength in LTRs. Circulating citrate may represent a potential metabolic biomarker of post-transplant muscle impairment.

Sarcopenia is highly prevalent in patients with end-stage liver disease and is increasingly recognized as a clinically important determinant of outcomes in the transplant setting [21,22]. Importantly, liver transplantation does not necessarily reverse muscle impairment. Recent studies suggest that cirrhosis-related sarcopenia may persist after transplantation, and in some recipients muscle abnormalities may fail to recover and may even worsen over time [23]. In addition, low muscle mass after transplantation has been linked not only to adverse prognosis but also to post-transplant metabolic complications, including diabetes [24]. These findings underscore the importance of continued surveillance of muscle health in liver transplant recipients. However, the identification and assessment of sarcopenia in this population remain challenging because no single tool adequately reflects the full spectrum of muscle impairment, and the available methods differ in feasibility, accessibility, and the specific domains they capture [25]. Current concepts therefore favor an integrated assessment strategy that includes muscle mass, muscle strength, and physical performance [25]. In this context, metabolomic profiling may provide complementary insights, particularly because metabolomic markers may help identify subclinical risk before overt functional decline becomes apparent [26,27]. It should be noted that a formal sarcopenia diagnosis according to the European Working Group on Sarcopenia in Older People (EWGSOP2) criteria was not performed in the present study. Instead, we evaluated estimated muscle mass (via CERI), handgrip strength, and limited physical performance tests. This approach allows exploration of muscle-related deficits but limits direct extrapolation to clinically diagnosed sarcopenia.

Our study demonstrated an inverse association between plasma citrate levels and muscle-related parameters in LTRs, particularly estimated muscle mass and muscle strength. Citrate is a central intermediate of the TCA cycle and occupies a key position at the interface of mitochondrial oxidative metabolism, anabolic signaling, and lipid synthesis. Disturbances in citrate homeostasis may therefore reflect broader abnormalities in mitochondrial substrate handling and metabolic flexibility [28]. Several transplant-specific mechanisms may contribute to elevated circulating citrate levels. These include impaired mitochondrial oxidative phosphorylation in skeletal muscle and other tissues, potentially leading to citrate accumulation due to reduced TCA cycle consumption [15]. Additional mechanisms involve altered citrate export via ATP-citrate lyase signaling, which links mitochondrial metabolism to cytosolic lipid synthesis and anabolic pathways [29]. Immunosuppressant-associated metabolic dysfunction is also likely relevant, as commonly used agents in LTRs, such as calcineurin inhibitors (e.g., tacrolimus) and corticosteroids, can impair mitochondrial calcium uptake, respiration, and energy production [30]. Moreover, post-transplant hepatic and renal metabolic alterations warrant consideration, given the liver’s central role in citrate production and the kidney’s dominant role in citrate reabsorption and excretion. A growing body of literature supports mitochondrial dysfunction as a major contributor to muscle wasting through impaired oxidative phosphorylation, excess reactive oxygen species generation, defective mitophagy, and altered protein turnover [31,32,33]. However, given the cross-sectional design of the study, the direction of this association should be interpreted cautiously. Skeletal muscle is a major metabolic tissue and may contribute to systemic citrate homeostasis through substrate uptake and utilization [34]. Accordingly, reduced muscle mass or impaired muscle metabolic capacity could itself lead to diminished citrate disposal, thereby contributing to higher circulating citrate concentrations (i.e., reverse causation). In this sense, the observed inverse association may reflect not only a potential effect of metabolic dysfunction on muscle mass but also the possibility that loss of muscle tissue alters systemic citrate homeostasis. By contrast, a small study in healthy volunteers reported that higher circulating citric acid was associated with better physical performance and lower adiposity, which is directionally opposite to our findings [35]. Rather than suggesting a uniform biological relationship across populations, these discrepant findings indicate that the association between citrate and muscle-related outcomes is likely context-dependent and influenced by underlying health status, organ function, inflammatory burden, and physical activity.

Beyond its association with muscle mass, plasma citrate was also inversely associated with muscle strength. Mediation analyses showed that muscle mass accounted for around 40% of the association between plasma citrate and handgrip strength. These findings suggest an association between citrate dysregulation and impaired intrinsic muscle function, particularly diminished contractile capacity [36,37]. Because muscle contraction and the maintenance of skeletal muscle integrity are all highly ATP-dependent processes, disturbances in energy metabolism may directly impair muscle strength [38,39]. Because our study is cross-sectional, we cannot establish causality. The mediation findings should be viewed as exploratory and hypothesis-generating only. Whether elevated circulating citrate reflects a primary metabolic state affecting muscle strength independent of muscle quantity, or vice versa, remains speculative and requires confirmation in longitudinal and mechanistic studies.

Furthermore, several factors might affect both citrate levels and muscle mass and strength and thereby contribute to the associations identified in the present study. First, a potential mechanism could be underlying inflammation, which may be related to both circulating citrate concentrations and muscle health. Chronic low-grade inflammation is known to promote muscle catabolism and impair muscle function [40], and it may also be accompanied by alterations in systemic metabolism [41]. However, adjustment for hs-CRP and GlycA did not materially affect the associations between plasma citrate levels and muscle parameters, suggesting that the observed associations are unlikely to be explained solely by systemic inflammation as captured by these markers. Second, impaired renal function may have played a role. Because the kidney plays an important role in citrate handling, reduced renal function may contribute to higher circulating citrate levels [16,42], while at the same time being associated with lower muscle mass and strength [43]. Nevertheless, the associations between plasma citrate levels and muscle parameters remained after adjustment for eGFR, indicating that differences in kidney function do not account for the observed relationships to a major extent. Another such factor may be BCAA metabolism, which is linked to TCA cycle-related energy metabolism and may therefore influence circulating citrate levels [44,45]. In our previous study, BCAA concentrations were positively associated with urinary creatinine excretion in LTRs [46]. However, adjustment for total BCAA concentrations did not alter the associations between citrate and muscle mass (CERI) or muscle strength. Taken together, the inverse association of plasma citrate with muscle mass and strength is unlikely to be explained solely by inflammation, renal function, or BCAA metabolism and may instead reflect broader metabolic disturbances relevant to impaired muscle health in liver transplant recipients.

This study has strengths and limitations. In addition to its novelty in LTRs, a major strength is the multidimensional assessment of muscle health, including measures of muscle mass, muscle strength, and physical performance, which provided a more comprehensive characterization of muscle status. Nevertheless, several limitations should be considered. First, because of the cross-sectional design, causal relationships cannot be established and reverse causation cannot be excluded. Second, information on dietary intake and physical activity was not available, and these unmeasured factors may influence muscle mass, mitochondrial metabolism, and circulating metabolites, including citrate. Physical inactivity in particular could plausibly contribute to both higher citrate concentrations and poorer muscle outcomes. Consequently, residual confounding from these unmeasured lifestyle variables cannot be excluded. Third, muscle mass was estimated using CERI rather than other imaging-based methods. Although CERI is practical and has been validated in transplant populations, it may be influenced by renal function, dietary protein intake, and completeness of urine collection. We adjusted for eGFR in all multivariable models, and urine collection completeness was assessed following standard protocols, with participants carefully instructed to ensure proper understanding of the procedures. When excluding participants with eGFR < 60 mL/min, the association between plasma citrate and CERI remains. Fourth, the number of participants available for the physical performance tests (STS and TUG) was relatively small, which reduced statistical power. Associations with STS and TUG lost significance after full adjustment, raising the possibility of type II error. However, participants completing these tests did not differ systematically from non-participants in key characteristics. Finally, formal sarcopenia diagnosis was not performed due to lack of the full set of criteria data. Therefore, no formal predictive analyses were conducted to evaluate the clinical utility of plasma citrate as a biomarker for sarcopenia. In addition, as the study was conducted in a predominantly European population, caution is warranted when extrapolating these findings to other populations.

## 4. Materials and Methods

### 4.1. Patient Population

This study was conducted and reported in accordance with the Strengthening the Reporting of Observational Studies in Epidemiology (STROBE) guidelines [47]. Participants were recruited from the TransplantLines cohort, a large-scale, prospective observational study based at the University Medical Center Groningen (UMCG), the Netherlands (NCT03272841), which is described in detail elsewhere [48]. The study protocol was approved by the UMCG Medical Ethics Committee (METc 2014/077), and all participants provided written informed consent. The study was conducted in compliance with the Declaration of Helsinki [49].

For the current cross-sectional analysis, we included clinically stable LTRs who were at least one year post-transplantation. To ensure independence of observations, when multiple study visits > 1 year post-transplantation were available, only the first visit was included. Inclusion was restricted to participants with available data on both plasma citrate levels and CERI, resulting in a final study population of 501 LTRs. Among these participants, handgrip strength, the STS test, and the TUG test were performed in subsets of 422, 189, and 178 LTRs, respectively. Data collection was carried out between December 2016 and November 2025. A detailed flow chart illustrating the participant selection process is provided in Supplemental Figure S1.

### 4.2. Data Collection

During standardized outpatient visits, comprehensive clinical data and biological samples were obtained from all participants following established protocols [48]. Participants were instructed to continue their regular medication regimens prior to blood sampling. Patient information, including weight, height, BMI (calculated as weight divided by height squared), smoking status, alcohol consumption, medication use (glucose-lowering drugs, antihypertensive medication, immunosuppressants), and medical histories such as hypertension and diabetes (defined as fasting plasma glucose > 7.0 mmol/L, non-fasting plasma glucose > 11.1 mmol/L, a self-reported diagnosis, or the use of glucose-lowering drugs), was extracted from the TransplantLines Biobank [48].

### 4.3. Assessment of Muscle Mass, Strength, and Physical Performance

Muscle mass was estimated using the 24 h urinary creatinine excretion rate divided by height squared (creatinine excretion rate index, CERI). This was done to take account of body size [9,50]: CERI (mmol/24 h/m^2^) = creatinine excretion (mmol/24 h)/height^2^ (m^2^). Participants were carefully instructed to ensure accurate urine collection, and any collections deemed incomplete were excluded from the primary analysis.

Handgrip strength was measured using a Jamar Hydraulic Hand Dynamometer (Patterson Medical JAMAR 5030J1, Warrenville, IL, USA). Participants performed three maximal attempts with each hand, with 30 s rest intervals between attempts. The highest value obtained from all attempts was used for analysis.

The STS and TUG tests were used to evaluate functional mobility and lower-limb performance. For the STS test, participants were instructed to rise from a chair and sit down five times as quickly as possible with their arms folded across the chest. After a practice trial, the test was performed three times, and the mean completion time (seconds) was used for analyses, with higher values indicating poorer performance. For the TUG test, participants were instructed to stand up from a chair without using their arms, walk at a usual pace to a marker 3 m away, turn around, walk back, and sit down. Walking aids were permitted if needed in accordance with standard protocol. After a practice round, the test was performed three times, and the mean time (seconds) was used for analyses, with higher values indicating poorer performance.

### 4.4. Laboratory Measurements

Fasting venous blood samples and 24 h urine samples were collected and analyzed according to standardized laboratory procedures. A comprehensive panel of assays, including serum alanine aminotransferase (ALT), AST, gamma-glutamyl transferase (GGT), ALP, albumin, creatinine, hemoglobin, glycated hemoglobin (HbA1c), plasma glucose, total cholesterol, and triglycerides, was performed at the Department of Laboratory Medicine, UMCG, using standard practice laboratory methods [48]. The eGFR was calculated using the 2021 Chronic Kidney Disease Epidemiology Collaboration (CKD-EPI) creatinine-based formula [51]. hs-CRP was measured via nephelometry (BNII; Dade Behring Diagnostics, Marburg, Germany) with a detection threshold of 0.18 mg/L. 24 h urinary creatinine excretion was calculated as the average of two consecutive 24 h urine collections following a standardized protocol [7].

For metabolic profiling, ethylenediaminetetraacetic acid (EDTA)-anticoagulated plasma samples were centrifuged at 1400× *g* for 15 min at 4 °C and then stored at −80 °C. Plasma samples were sent frozen at −80 °C to Labcorp (Morrisville, NC, USA) for analysis on the Vantera^®^ Clinical Analyzer (Labcorp Inc., Raleigh, NC, USA). Citrate levels were determined using NMR spectroscopy as previously described [17,18,20]. Inter-assay precision for citrate, expressed as coefficients of variation (%CV), ranged from 5.2% for high-concentration samples to 9.6% for low-concentration samples. Plasma concentrations of total BCAA and GlycA (a marker of systemic inflammation reflecting N-acetyl methyl groups of glycoproteins) were quantified using established NMR-based algorithms as previously described [47,52,53].

### 4.5. Statistical Analyses

Statistical analyses were performed using IBM SPSS Statistics (version 25.0; IBM Corp., Armonk, NY, USA) and R (version 4.2.1; R Foundation for Statistical Computing, Vienna, Austria). A two-sided *p*-value < 0.05 was considered statistically significant.

Continuous variables are presented as median (IQR) and categorical variables as numbers (percentages). Differences in baseline characteristics across plasma citrate quartiles were assessed using the Kruskal–Wallis test for continuous variables and the χ^2^ test or Fisher’s exact test, as appropriate, for categorical variables.

Spearman’s rank correlation was used to assess univariable correlations among muscle-related parameters (CERI, handgrip strength, STS time, and TUG time) and to evaluate associations between plasma citrate and these parameters. Associations between log_2_-transformed plasma citrate (continuous exposure) and estimated muscle mass/physical performance outcomes were further examined using linear regression models, reporting standardized regression coefficients (Std. β) with 95% confidence intervals (CI). A priori defined regression models were built sequentially as follows: Model 1, unadjusted; Model 2, adjusted for age, sex, and BMI; Model 3, additionally adjusted for alcohol intake, hypertension, diabetes, eGFR, AST, hs-CRP, GlycA and BCAA; and Model 4, additionally adjusted for the use of calcineurin inhibitors, antimetabolite agents, and time since transplantation. Potential effect modification by sex, age, and diabetes status was assessed by including multiplicative interaction terms in the fully adjusted model.

To complement analyses of continuous outcomes, logistic regression models were performed using dichotomized muscle-related endpoints. Low handgrip strength was defined according to the EWGSOP2 criteria (men < 27 kg and women < 16 kg) [54]. For CERI and performance-based measures (STS and TUG), no established clinical cut-offs are currently available. Therefore, these variables were dichotomized at the sample median to allow relative risk comparisons within the study population. Odds ratios (ORs) and 95% CIs were calculated across citrate quartiles, with Q1 as the reference group.

Mediation analysis was performed to evaluate whether the associations between plasma citrate and physical function (handgrip strength, STS, and TUG) were mediated by muscle mass (CERI) using the “mediation” package in R. Direct, indirect, and total effects were estimated using nonparametric bootstrap resampling (1000 iterations) to derive 95% CIs. Height-adjusted handgrip strength (maximal grip strength divided by height squared) was additionally used in sensitivity analyses.

## 5. Conclusions

In conclusion, higher plasma citrate levels were independently associated with lower estimated muscle mass and muscle strength in liver transplant recipients. These findings highlight a potential link between citrate metabolism and impaired muscle status in this population. However, due to the absence of dietary and physical activity data, residual confounding cannot be excluded, and circulating citrate should be considered a potential rather than established independent biomarker. Further longitudinal and mechanistic studies with comprehensive lifestyle assessment are needed to clarify the temporal and causal nature of these associations and to determine whether circulating citrate may provide additional value in risk stratification.

## Figures and Tables

**Table 1:** Baseline characteristics of 501 liver transplant recipients.

	All	Quartiles of Plasma Citrate Levels				*p*-Value
		Q1 (<101 µmol/L)	Q2 (101–121 µmol/L)	Q3 (121–148 µmol/L)	Q4 (>148 µmol/L)	
Number of participants	501	124	123	127	127	
Age, years	59 (49, 67)	53 (40, 62)	60 (48, 67)	61 (50, 67)	60 (54, 68)	<0.001
Men, *n* (%)	294 (58.7)	79 (63.7)	85 (69.1)	60 (47.2)	70 (55.1)	0.002
Body mass index, kg/m^2^	26.2 (23.4, 30.1)	25.6 (23.2, 29.6)	26.4 (23.4, 29.4)	25.5 (23.0, 30.0)	27.5 (23.8, 31.2)	0.030
Alcohol intake, units/week						0.003
None, *n* (%)	412 (82.2)	95 (76.6)	94 (76.4)	103 (81.1)	120 (94.5)	
0–7 units per week, *n* (%)	61 (12.2)	20 (16.1)	20 (16.3)	18 (14.2)	3 (2.4)	
>7 units per week, *n* (%)	28 (5.6)	9 (7.3)	9 (7.3)	6 (4.7)	4 (3.1)	
Current smoking, *n* (%)	47 (9.4)	7 (5.6)	12 (9.8)	16 (12.6)	12 (9.4)	0.308
Hypertension, *n* (%)	271 (54.1)	50 (40.3)	60 (48.8)	72 (56.7)	89 (70.1)	<0.001
Diabetes, *n* (%)	129 (25.7)	26 (21.0)	27 (22.0)	35 (27.6)	41 (32.3)	0.141
Oral glucose-lowering drugs, *n* (%)	55 (11.0)	7 (5.6)	8 (6.5)	15 (11.8)	25 (19.7)	0.001
Insulin, *n* (%)	69 (13.8)	14 (11.3)	13 (10.6)	17 (13.4)	25 (19.7)	0.141
Antihypertensives, *n* (%)	271 (54.1)	50 (40.3)	60 (48.8)	72 (56.7)	89 (70.1)	<0.001
Laboratory measurements						
ALT (U/L)	29.0 (21.0, 44.0)	32.0 (22.8, 56.2)	28.0 (18.0, 44.0)	27.0 (20.0, 35.0)	29.5 (23.0, 43.8)	0.073
AST (U/L)	31.0 (23.0, 48.0)	28.0 (21.8, 50.5)	26.0 (21.0, 43.0)	29.0 (23.2, 37.0)	39.0 (31.0, 58.5)	<0.001
GGT (U/L)	57.0 (28.2, 131.5)	63.5 (27.8, 168.2)	58.0 (28.0, 114.0)	46.0 (26.0, 101.5)	66.0 (36.5, 140.5)	0.099
ALP (U/L)	99.0 (75.0, 164.0)	98.0 (74.8, 177.0)	90.0 (69.0, 144.0)	91.0 (77.0, 157.2)	122.0 (87.8, 174.2)	0.005
Albumin (g/L)	43.0 (37.0, 45.0)	43.0 (39.8, 46.0)	44.0 (41.0, 46.0)	43.0 (40.0, 46.0)	35.5 (30.0, 43.0)	<0.001
Total cholesterol (mmol/L)	4.2 (3.4, 5.0)	4.3 (3.5, 4.9)	4.5 (3.8, 5.4)	4.5 (3.7, 5.3)	3.7 (3.1, 4.5)	<0.001
Triglycerides (mg/dL)	1.1 (0.8, 1.6)	1.3 (0.8, 1.6)	1.1 (0.8, 1.7)	1.2 (0.9, 1.8)	1.0 (0.7, 1.3)	<0.001
Fasting glucose (mmol/L)	5.8 (5.2, 7.3)	5.7 (5.2, 7.4)	5.8 (5.2, 6.9)	5.6 (5.1, 6.9)	6.0 (5.2, 7.6)	0.516
HbA1c (%)	5.4 (5.0, 5.9)	5.3 (4.9, 5.8)	5.5 (5.0, 6.1)	5.4 (5.1, 5.9)	5.4 (4.8, 6.0)	0.124
Hemoglobin (mmol/L)	8.1 (7.2, 9.1)	8.1 (7.1, 9.2)	8.5 (7.7, 9.3)	8.2 (7.3, 9.1)	7.6 (6.7, 8.4)	<0.001
Serum creatinine (μmol/L)	87.0 (71.0, 110.0)	81.0 (65.0, 103.0)	89.0 (74.0, 115.0)	89.0 (74.0, 111.5)	87.0 (70.0, 104.0)	0.067
eGFR, mL/min/1.73 m^2^	78.8 (60.3, 99.7)	85.7 (70.2, 108.2)	78.0 (58.7, 96.1)	75.5 (56.9, 97.2)	78.6 (63.0, 96.6)	0.003
Urinary creatinine excretion, mmol/24 h	10.9 (8.5, 14.2)	12.2 (9.4, 16.0)	12.0 (9.6, 15.1)	10.1 (8.3, 12.9)	10.3 (8.0, 12.2)	<0.001
hs-CRP, mg/L	3.3 (1.3, 9.0)	3.6 (1.2, 13.0)	2.8 (1.1, 8.0)	2.9 (1.3, 6.0)	4.3 (2.1, 12.0)	0.026
Total BCAA, µmol/L	326.0 (275.0, 390.0)	350.8 (296.2, 424.5)	330.0 (289.9, 400.5)	322.0 (270.0, 374.5)	301.0 (258.0, 348.0)	<0.001
GlycA, µmol/L	358.3 (304.0, 429.0)	380.0 (329.8, 473.2)	385.0 (327.5, 430.0)	355.0 (319.0, 423.5)	318.0 (255.1, 383.5)	<0.001
Immunosuppressants						
Calcineurin inhibitors, *n* (%)	253 (50.5)	73 (58.9)	59 (48)	82 (64.6)	39 (30.7)	<0.001
Antimetabolite agents, *n* (%)	195 (38.9)	50 (40.3)	57 (46.3)	53 (41.7)	35 (27.6)	0.016
Glucocorticoids, *n* (%)	189 (37.7)	45 (36.3)	58 (47.2)	47 (37)	39 (30.7)	0.059
mTOR inhibitors, *n* (%)	44 (8.8)	14 (11.3)	10 (8.1)	11 (8.7)	9 (7.1)	0.683
Time since transplantation, years	14.0 (7.4, 22.5)	11.4 (7.3, 20.5)	17.0 (10.9, 24.8)	14.5 (8.4, 22.6)	9.0 (5.8, 19.8)	0.002
Muscle mass and strength parameters						
CERI, mmol/24 h/m^2^	3.7 (2.9, 4.5)	4.1 (3.2, 4.9)	3.9 (3.1, 4.7)	3.5 (2.9, 4.2)	3.5 (2.8, 4.1)	<0.001
Handgrip strength, kg	33.3 (25.9, 42.3)	36.8 (27.9, 46.5)	36.3 (28.5, 44.5)	32.2 (24.3, 40.8)	30.5 (24.2, 38.5)	<0.001
Sit-to-stand test, s	12.0 (9.6, 15.0)	11.3 (8.7, 13.5)	12.0 (9.7, 15.0)	12.2 (9.9, 14.9)	13.3 (10.4, 16.6)	0.082
Timed Up and Go test, s	7.2 (6.2, 8.4)	7.0 (5.7, 8.1)	6.8 (6.2, 8.1)	7.2 (6.5, 7.9)	8.0 (6.7, 9.2)	0.040

Data are presented as median (IQR) or as proportions (*n*) with corresponding percentages (%). Abbreviations: ALT: alanine aminotransferase; AST: aspartate aminotransferase; GGT: gamma-glutamyl transferase; ALP: alkaline phosphatase; HbA1c: hemoglobin A1c; eGFR: estimated glomerular filtration rate; hs-CRP: high-sensitivity C-reactive protein; BCAA: branched-chain amino acids; CERI: 24 h urinary creatinine excretion rate index. Data on handgrip strength were available for 422 LTRs. The sit-to-stand test was performed in 189 LTRs. The Timed Up and Go (TUG) test was performed in 178 LTRs.

**Table 2:** Linear regression of the association between citrate levels with different measurements of muscle mass, strength, and physical performance.

	Model 1		Model 2		Model 3		Model 4	
	Std. β	*p*-Value	Std. β	*p*-Value	Std. β	*p*-Value	Std. β	*p*-Value
Creatinine excretion rate index, mmol/24 h/m^2^ *n* = 501	−0.19 (−0.28, −0.10)	<0.001	−0.14 (−0.22, −0.06)	0.001	−0.11 (−0.21, −0.02)	0.014	−0.14 (−0.23, −0.05)	0.001
Handgrip strength, kg *n* = 422	−0.19 (−0.29, −0.10)	<0.001	−0.10 (−0.17, −0.03)	0.005	−0.14 (−0.22, −0.06)	0.001	−0.11 (−0.20, −0.03)	0.010
Sit-to-stand test, s *n* = 189	0.22 (0.07, 0.37)	0.005	0.16 (0, 0.31)	0.053	0.09 (−0.09, 0.27)	0.346	0.10 (−0.08, 0.28)	0.265
Timed Up and Go test, s *n* = 178	0.28 (0.12, 0.44)	0.001	0.20 (0.05, 0.36)	0.012	0.13 (−0.06, 0.31)	0.185	0.11 (−0.08, 0.30)	0.249

Model 1: Crude. Model 2: Adjusted for age, sex, and BMI. Model 3: As in Model 2, with additional adjustments for alcohol intake, hypertension, diabetes, eGFR, AST, hs-CRP, GlycA and BCAA. Model 4: As in Model 3, with additional adjustments for the use of calcineurin inhibitors, antimetabolite agents, and time since transplantation.

## Data Availability

Data are available upon reasonable request from the corresponding author due to privacy restrictions.

## Supplementary Materials

The following supporting information can be downloaded at: https://www.mdpi.com/article/10.3390/ijms27114809/s1.

## Abbreviations

The following abbreviations are used in this manuscript:

LTR	Liver transplant recipients
TCA	Tricarboxylic acid
NMR	Nuclear magnetic resonance
CERI	Creatinine excretion rate index
STS	Sit-to-Stand
TUG	Timed Up and Go
BMI	Body mass index
AST	Aspartate aminotransferase
ALP	Alkaline phosphatase
eGFR	Estimated glomerular filtration rate
hs-CRP	High-sensitivity C-reactive protein
BCAA	Branched-chain amino acids
ALT	Alanine aminotransferase
GGT	Gamma-glutamyl transferase
EDTA	Ethylenediaminetetraacetic acid
CV	Coefficients of variation
CI	Confidence interval
