# Supplementary material for: Plasma Citrate Levels Are Inversely Associated with Estimated Muscle Mass and Strength in Liver Transplant Recipients
Source: Int J Mol Sci. 2026 May 27;27(11):4809. doi: 10.3390/ijms27114809 (PMC13256401; doi:10.3390/ijms27114809)
Supplement: Supplementary file 1 [file ijms-27-04809-s001.zip › ijms-4310319-supplementary.pdf]

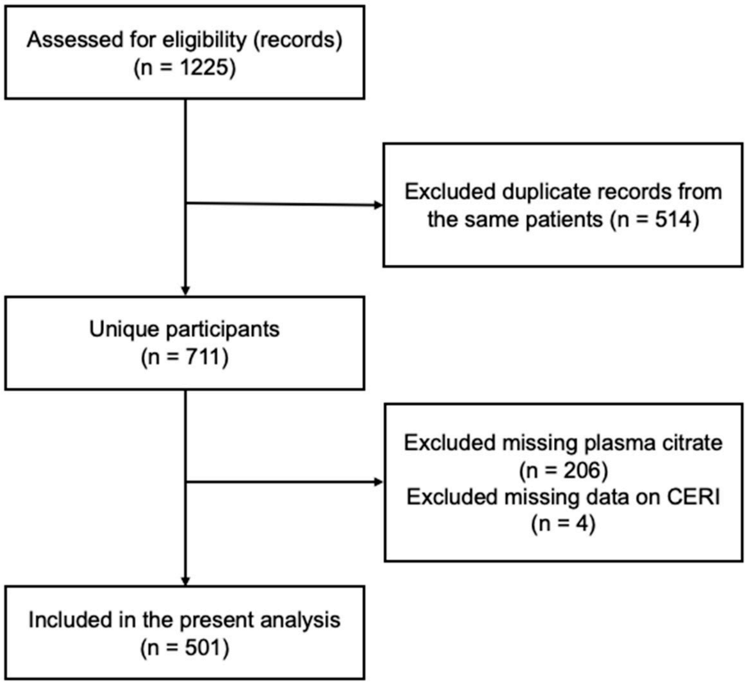

Supplemental Figure S1 Flow chart illustrates the participant selection process. CERI: 24-h urinary creatinine excretion rate index

Supplementary Table S1 Association between quartiles of citrate levels and the odds of below-median muscle mass and strength measures

	**Below-median CERI**		**Low handgrip strength**		**Above-median STS time**		**Above-median TUG time**	
	OR (95% CI)	P value	OR (95% CI)	P value	OR (95% CI)	P value	OR (95% CI)	P value
Univariable								
Quartile 1	Reference		Reference		Reference		Reference	
Quartile 2	1.29 (0.77, 2.15)	0.336	1.37 (0.42, 4.47)	0.6	1.08 (0.5, 2.37)	0.842	1.09 (0.48, 2.45)	0.837
Quartile 3	**2.79 (1.68, 4.66)**	**<0.001**	0.53 (0.12, 2.27)	0.392	1.38 (0.62, 3.05)	0.433	1.46 (0.65, 3.32)	0.362
Quartile 4	**2.89 (1.73, 4.82)**	**<0.001**	**3.12 (1.08, 9.02)**	**0.036**	1.98 (0.85, 4.58)	0.112	**3.78 (1.5, 9.53)**	**0.005**
Multivariable								
Quartile 1	Reference		Reference		Reference		Reference	

Quartile 2	1.44	(0.74, 2.81)	0.288	1.05	(0.3, 3.69)	0.943	0.77	(0.3, 2.02)	0.6	0.66	(0.24, 1.77)	0.404
Quartile 3	**2.51**	**(1.3, 4.85)**	**0.006**	0.45	(0.1, 2.09)	0.309	1	(0.39, 2.55)	0.994	0.96	(0.36, 2.56)	0.928
Quartile 4	**2.1**	**(1.06, 4.19)**	**0.034**	2.44	(0.72, 8.29)	0.152	0.93	(0.32, 2.67)	0.89	1.13	(0.36, 3.54)	0.831

CERI: 24-h urinary creatinine excretion rate index; STS: sit-to-stand; TUG: Timed up and go.

Multivariable model: Adjusted for age, sex, and BMI, eGFR, hs-CRP, calcineurin inhibitors, antimetabolite agents, and time since transplantation.
